# Supplementary material for: Coherent vibrational dynamics reveals lattice anharmonicity in organic–inorganic halide perovskite nanocrystals
Source: Nat Commun. 2021 May 11;12:2629. doi: 10.1038/s41467-021-22934-2 (PMC8113605; doi:10.1038/s41467-021-22934-2)
Supplement: Supplementary file 1 — Supplementary Information [file 41467_2021_22934_MOESM1_ESM.pdf]

Coherent Vibrational Dynamics Reveals Lattice Anharmonicity in Organic-inorganic Halide Perovskite Nanocrystals

Debnath et al.

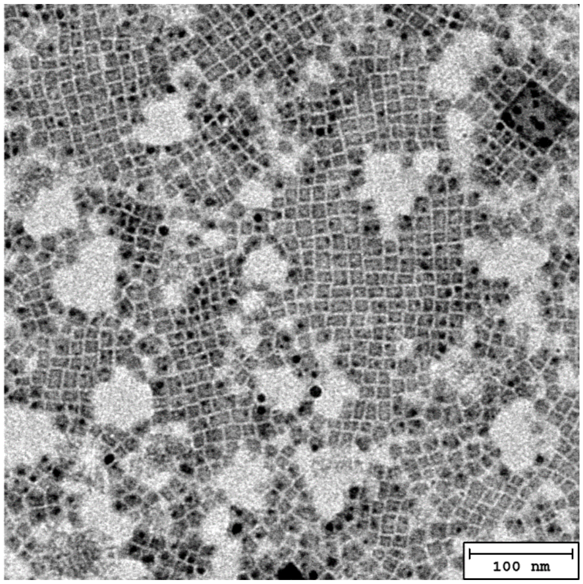

Supplementary Figure 1. Large-area overview transmission electron microscopy (TEM) image of the pristine $FAPbBr_3$ PNCs.

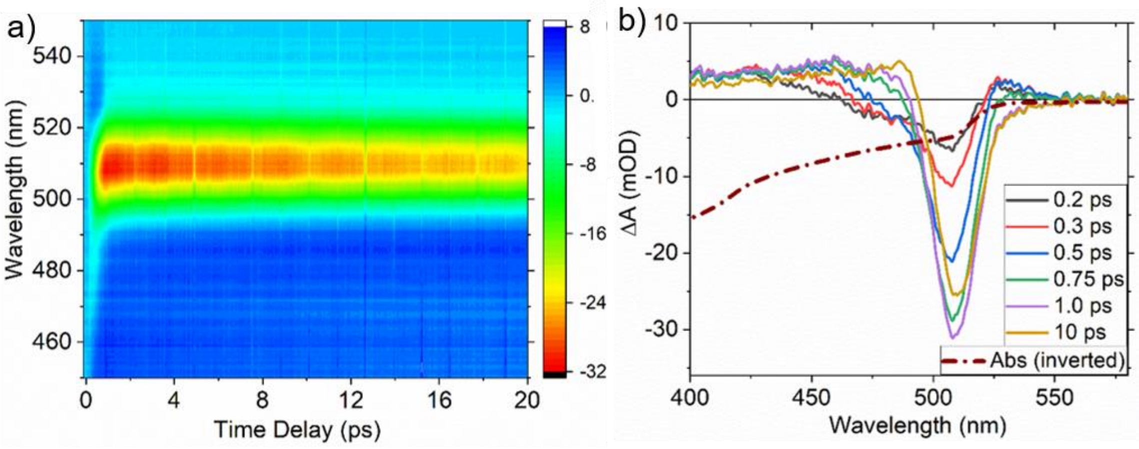

Supplementary Figure 2. Transient differential absorption (ΔA) spectra of the $FAPbBr_3$ PNCs as measured (before data processing) depicted in a) a contour diagram (showing significant chirp) and b) at different time delay (0.2 ps to 10 ps), excited with a ~100 fs laser pulse. The corresponding steady-state absorption spectrum (inverted) is plotted for comparison in the maroon dot-dashed line.

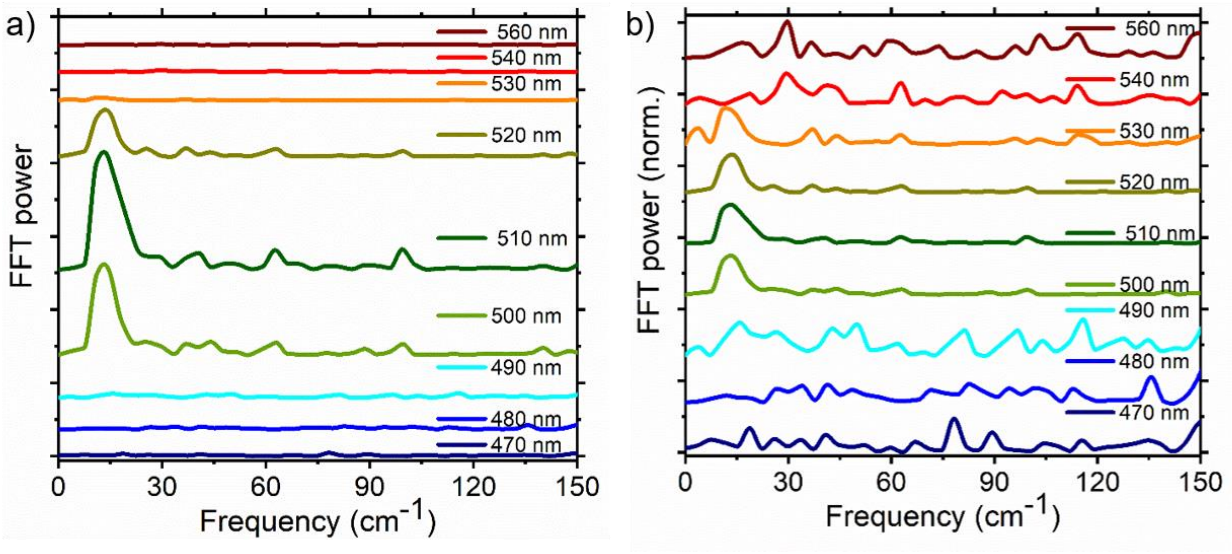

Supplementary Figure 3. The FFT power spectra of the $FAPbBr_3$ PNCs at different probe wavelengths (blue to red region), computed over the first 10 ps time delay. In a), all the spectra are plotted in the same intensity scale (for comparison) and in b) all the spectra are normalized to maximum and finally, each spectrum is vertically shifted for clarity.

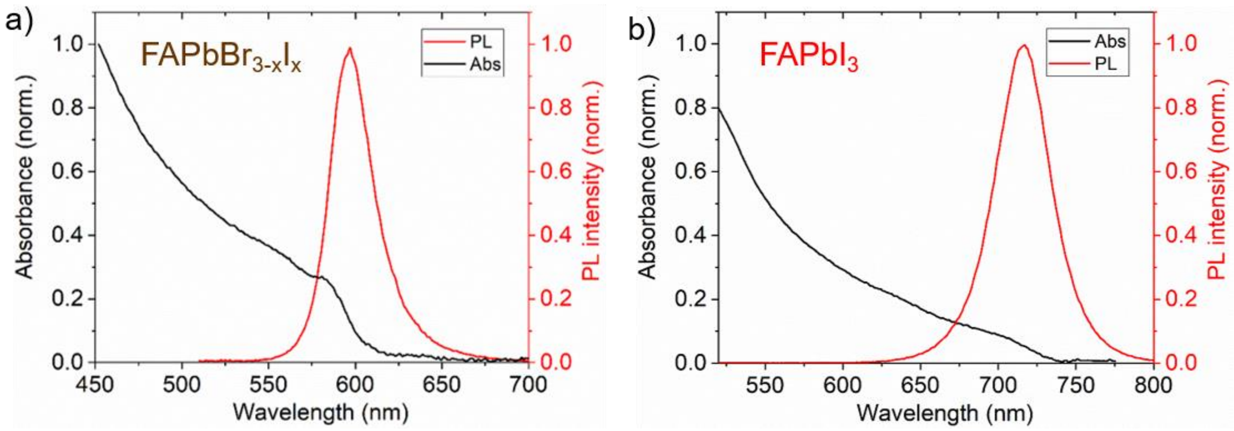

Supplementary Figure 4. Steady-state optical absorption and PL spectra of the a) $FAPbBr_{3-x}I_x$ and b) $FAPbI_3$ PNCs, prepared by ion-exchanged method.

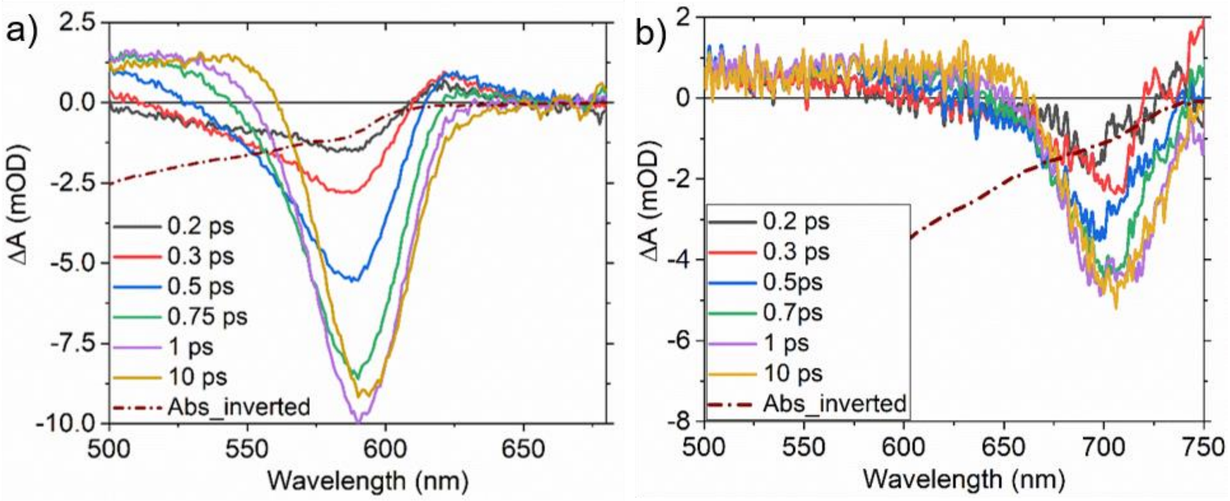

Supplementary Figure 5. Transient differential absorption (ΔA) spectra at different time delay (0.2 ps to 10 ps) of a) $FAPbBr_{3-x}I_x$ and b) $FAPbI_3$ PNCs, excited with a ~100 fs laser pulse. The steady-state absorption spectrum (inverted) of each PNCs, are plotted for comparison in the maroon dot-dashed line.

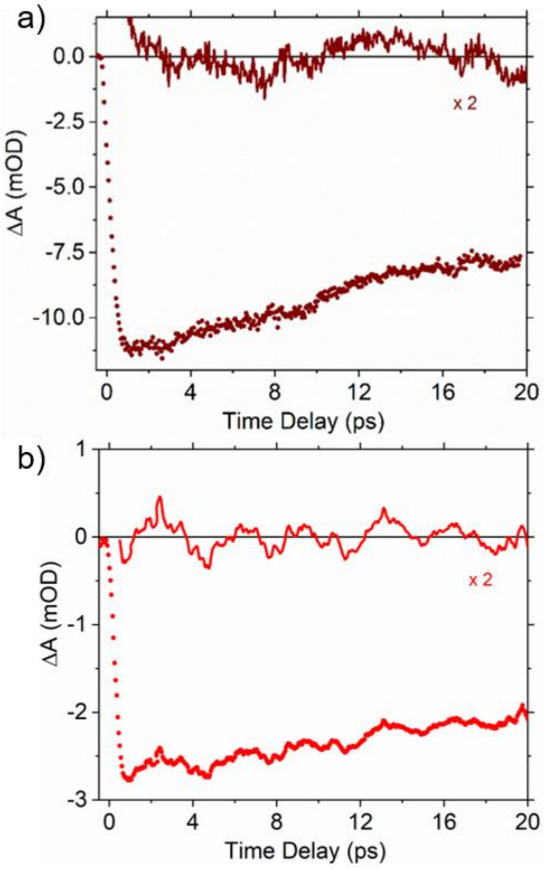

Supplementary Figure 6. Time trace obtained a) at 590 nm for $FAPbBr_{3-x}I_x$ and b) at 690 nm for $FAPbI_3$ PNCs. The residual time trace, obtained after subtraction of the exponential fit, is also shown as a maroon and red line respectively, for both the PNCs.

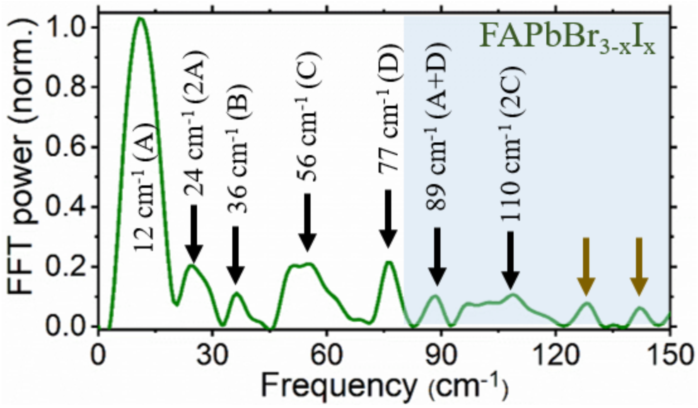

Supplementary Figure 7. The FFT power spectrum of the $FAPbBr_{3-x}I_x$ PNCs at 590 nm, computed over the first 10 ps time delay. The peaks indicated by dark-yellow arrow are due to FA-librational modes.

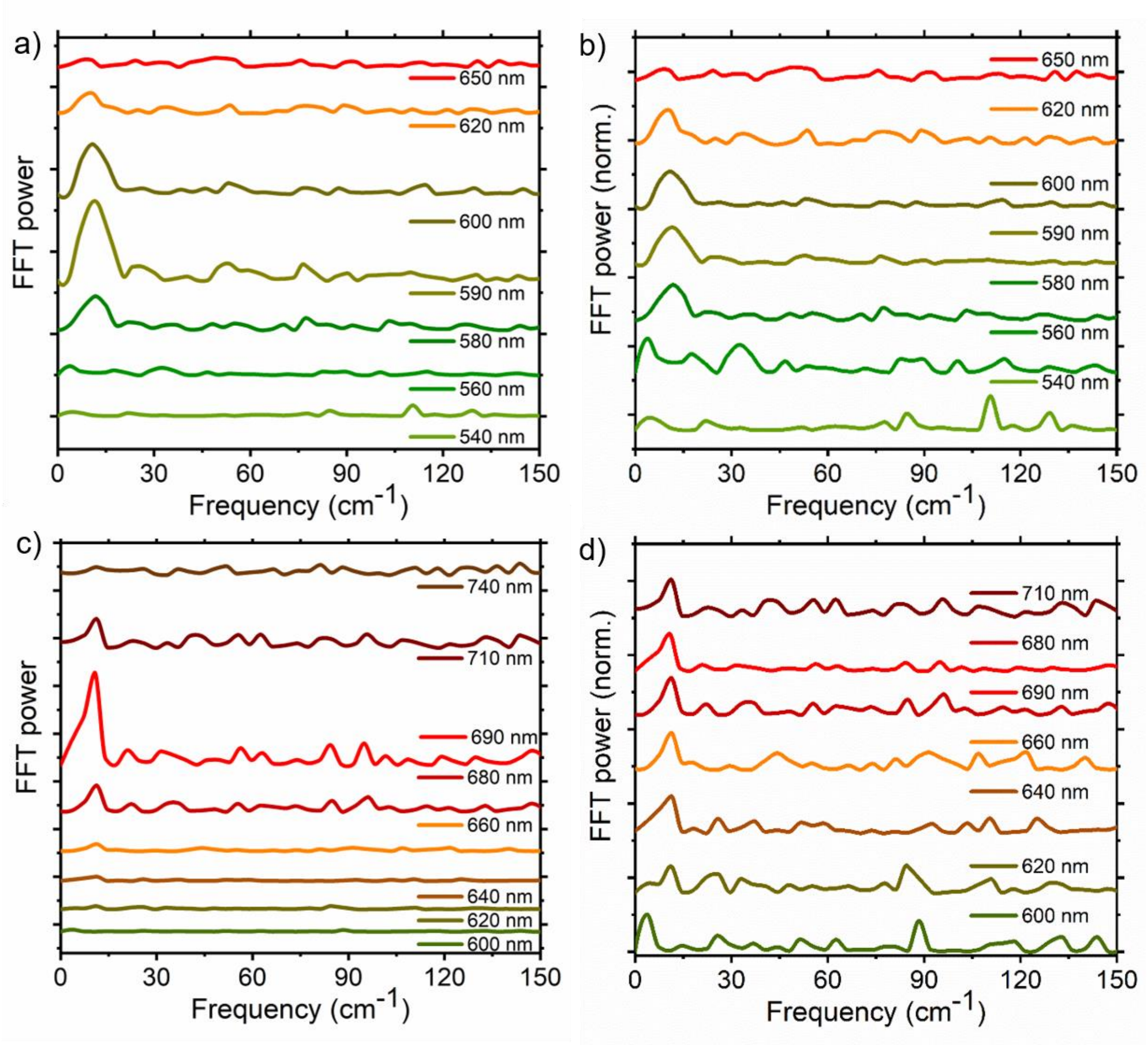

Supplementary Figure 8. The FFT power spectra of the a, b) $FAPbBr_{3-x}I_x$ and c, d) $FAPbI_3$ PNCs at different probe wavelengths, computed over the first 10 ps time delay. In a) and c), all the spectra are plotted in the same intensity scale (for comparison) and in b) and d) all the spectra are normalized to maximum and finally, each spectrum is vertically shifted for clarity.

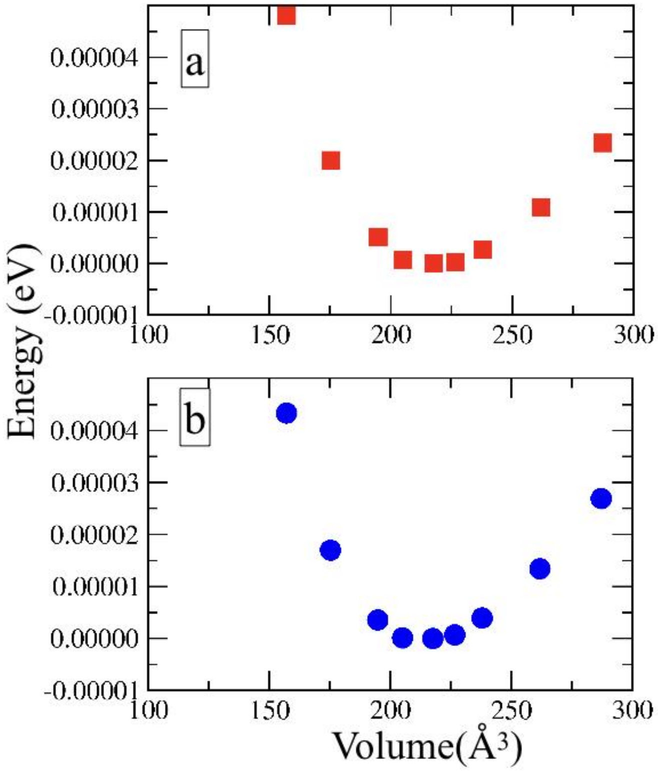

Supplementary Figure 9. The energy variation as a function of unit cell volume in $FAPbBr_3$ a) without SOC and b) including SOC.

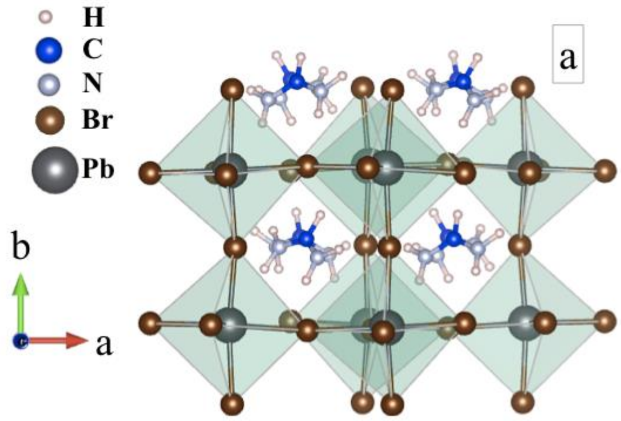

Supplementary Figure 10. 2x2x2 supercell structure of pristine $FAPbBr_3$ showing the different equilibrium orientations of FA moieties in different cages.

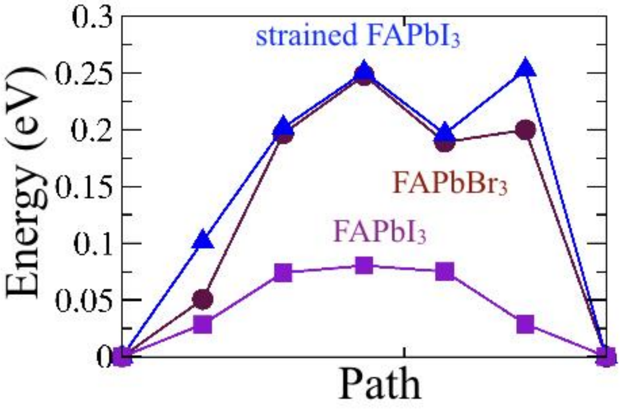

Supplementary Figure 11. Calculated minimum-energy path for the rotation of FA moiety between two stable symmetrically equivalent configurations. The results are shown for $FAPbI_3$ (purple square), constrained $FAPbI_3$ (blue triangle) and $FAPbBr_3$ (maroon circle).

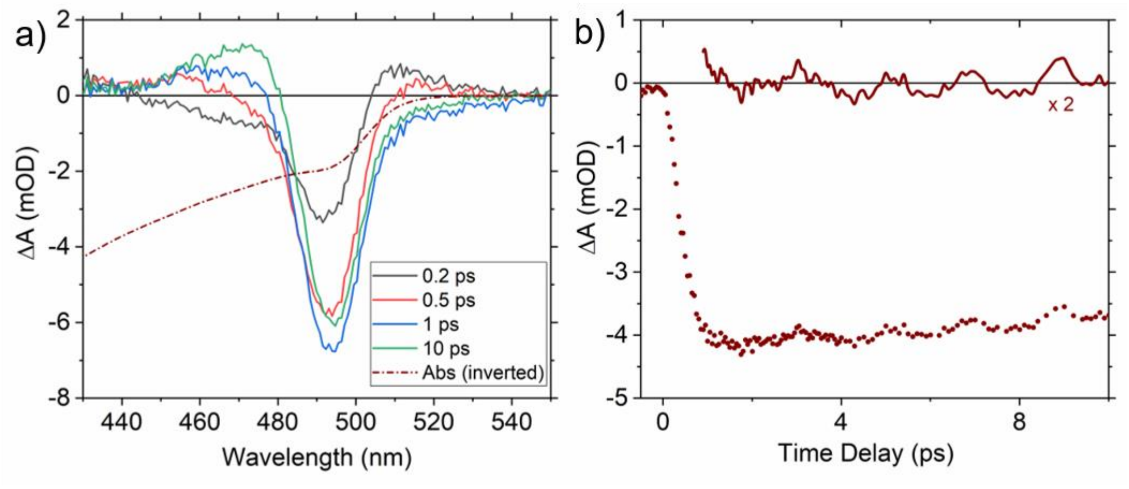

Supplementary Figure 12. a) Transient differential absorption (ΔA) spectra at different time delay (0.2 ps to 10 ps) of $MAPbBr_3$ PNCs, excited with a ~100 fs laser pulse. The steady-state absorption spectrum (inverted) is also plotted for comparison in the maroon dot-dashed line. b) Time trace obtained at 500 nm for $MAPbBr_3$ PNCs. The residual time trace, obtained after subtraction of the exponential fit, is also shown as a maroon line the PNCs.

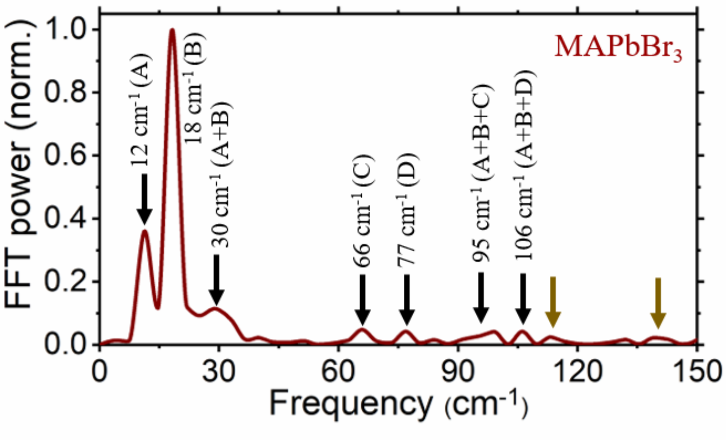

Supplementary Figure 13. The FFT power spectrum of the $MAPbBr_3$ PNCs at 500 nm, computed over the first 10 ps time delay. The peaks indicated by dark-yellow arrows are due to MA-librational modes.

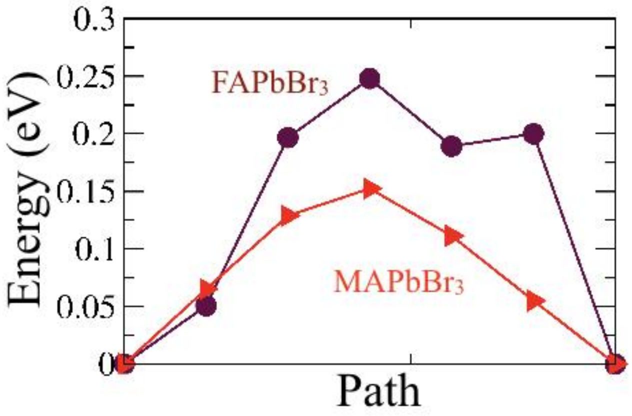

Supplementary Figure 14. Calculated minimum-energy path for the rotation of MA and FA moiety between two stable symmetrically equivalent configurations in $MAPbBr_3$ (red triangle) and $FAPbBr_3$ (maroon circle).

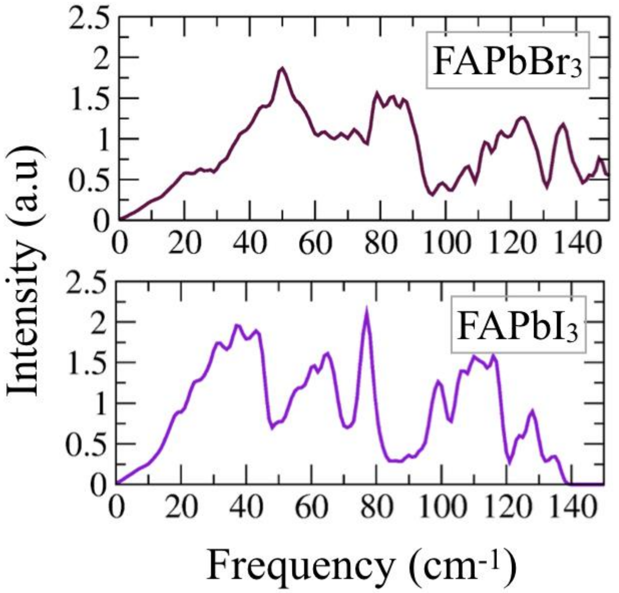

Supplementary Figure 15. Phonon density of states at the Γ point for 2x2x2 supercell of $FAPbBr_3$, and $FAPbI_3$.

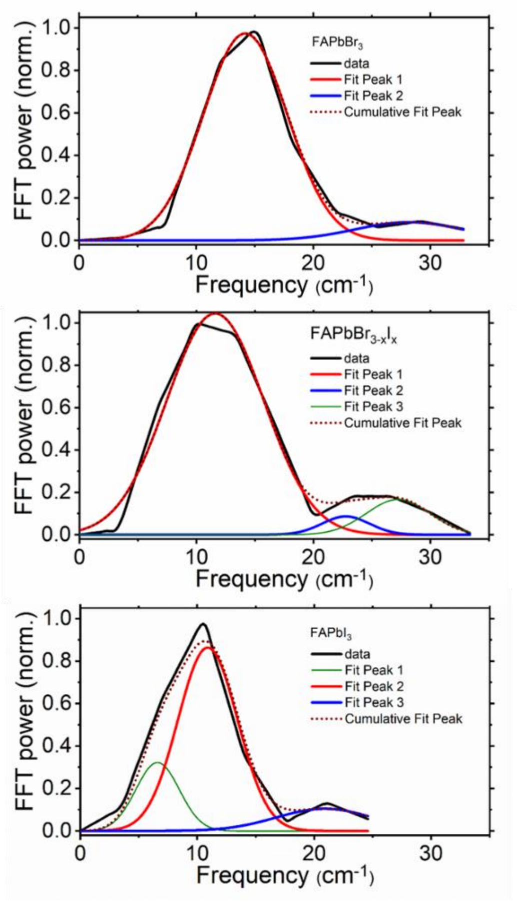

Supplementary Figure 16. Gaussian fitting of A and 2A modes of the FFT power spectrum of $FAPbBr_3$, $FAPbBr_{3-x}I_x$ and $FAPbI_3$ PNCs (top to bottom).

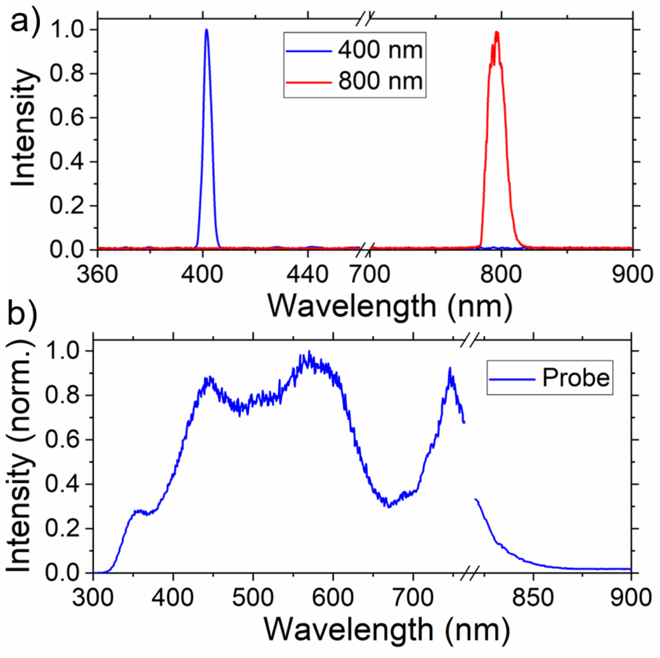

Supplementary Figure 17. a) The 800 nm laser and 400 nm pump spectrum. b) The continuum white light probe spectrum.

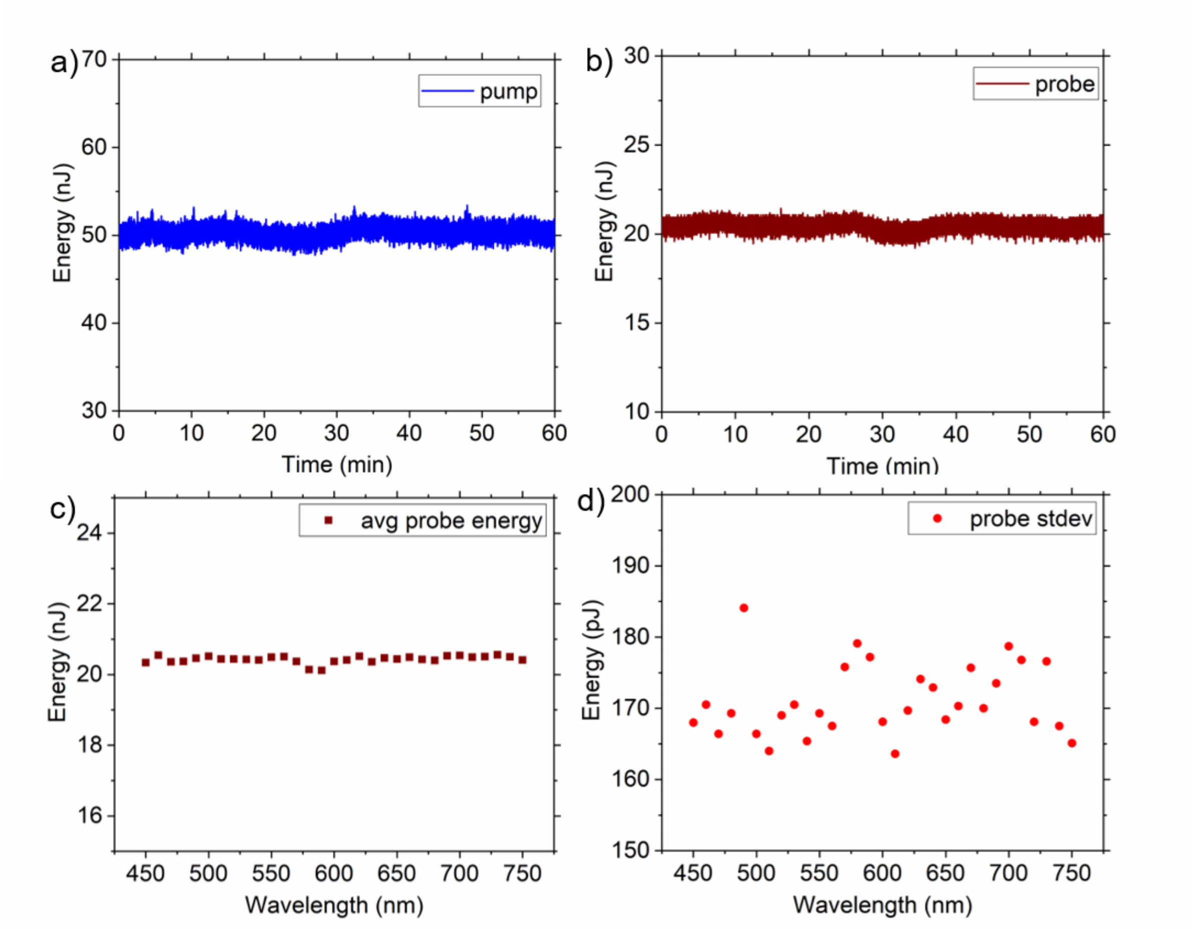

Supplementary Figure 18. The energy stability of a) the 400 nm pump and b) the continuum white light probe pulse (at 600 nm), measured for 1 h duration. c) The average energy (in nJ) of the attenuated continuum white light probe pulse between 450 nm to 750 nm, measured for 60 sec each, in the span of total ~1 h duration and d) the corresponding wavelength dependent standard deviation (in pJ) of the probe pulse.

Supplementary Table 1. Experimental vibrational frequencies and their assignments of the colloidal $FAPbBr_3$, $FAPbBr_xI_{3-x}$, and $FAPbI_3$ PNCs.

	Frequency (cm^{-1})			**Assignment**
	**$FAPbBr_3$ λ_{abs}~510 nm**	**$FAPbBr_xI_{3-x}$ λ_{abs} ~590nm**	**$FAPbI_3$ λ_{abs} ~690nm**	
	Expt.	Expt.	Expt.	
1	15	12	11	Pb-X bending (A)
2	29	24	21	2A
3	38	36	31	Pb-X bending (B)
4	62	56	53	Pb-X stretching (C)
5		77	60	Pb-X stretching (D)
6	98		81	B+C
7		89		A+D
8			91	B+D
9		110		2C
10			120	2D
11	140	129, 144	111, 130, 147	FA-librational

Supplementary Note 1: Preparation of $FAPbBr_{3-x}I_x$ and $FAPbI_3$ perovskite nanocrystals (PNCs)

$FAPbBr_{3-x}I_x$ and $FAPbI_3$ PNCs were prepared by ion-exchange method from the parent $FAPbBr_3$ PNCs.[1] For this, PbI_2 solution was prepared by dissolving 0.1 mmol PbI_2 powder in a mixture of 100 µl of oleic acid, 100 µl oleylamine, and 10 mL toluene (concentration: ~0.01 mol/L) at 80 °C under continuous stirring for overnight. By adding different amounts of PbI_2 solution, different ratio of Br and I in $FAPbBr_{3-x}I_x$ PNCs can be obtained. To obtain the desired absorption of $FAPbBr_{3-x}I_x$ PNC, 10 µl of PbI_2 solution was added into the $FAPbBr_3$ PNC solution for few times and left it for 10-15 min to reach the equilibrium until the desired absorption was obtained. Thus prepared ion-exchanged $FAPbBr_{3-x}I_x$ and $FAPbI_3$ PNCs were used for further experiments. It should be mentioned, when further addition of the PbI_2 solution to the parent $FAPbBr_3$ PNCs didn't cause a shift in the absorption and the photoluminescence (PL) position, it was considered as $FAPbI_3$ PNCs.

Supplementary Note 2: Preparation of $MAPbBr_3$ PNCs

To prepare the $MAPbBr_3$ PNCs, MABr (0.32 mmol), $PbBr_2$ (0.4 mmol) along with 0.1 mL oleylamine and 1 mL oleic acid were co-dissolved in 10 mL DMF.[2] 0.5 mL of the prepared precursor solution was quickly injected into 5 mL toluene at 60 °C under vigorous stirring. Strong PL observed immediate after injection due to the formation of $MAPbBr_3$ PNCs. The product was centrifuged to discard the unreacted species.

Supplementary Note 3: Reason and method of anharmonicity calculation along the strongest Pb-X bending coordinate

We considered only the strongest Pb-X bending coordinate for the anharmonicity calculation. This is because the ground-to-excited state displacement of the potential minima is maximized along the Pb-X bending, which are 14.7 cm^{-1} and 10.9 cm^{-1} for $FAPbBr_3$ and $FAPbI_3$, respectively. Therefore, these are predominately responsible for the excited state deformation. Please note that, all the frequency values are approximated to near integer values in the manuscript. The relative ground-to-excited state potential minima displacement can be determined for each Raman peak using the relation, $\sigma_R \propto \Delta^2\omega^2$.[3] Here σ_R, Δ

and ω are the Raman scattering cross-section, the ground-to-excited potential minima displacement and the vibrational frequency, respectively. Here, the relative displacement ratio is found to be ±10.4, ±1.7 and ±1.0 for A, B and C modes, respectively for $FAPbBr_3$.

The FFT spectral resolution is ~3.33 cm^{-1} for the ~10 ps temporal window in this work. Here we are interested in the frequency values <30 cm^{-1} for estimation of the anharmonicity constant using the Morse potential, however, our spectral resolution doesn't allow us to quantify the exact frequency values required. Therefore, we approximated the frequency values by modeling with Gaussian functions. Although the $FAPbBr_3$ PNCs can nicely be modelled by two Gaussians for A and 2A modes, for the other two PNCs a third Gaussian is required to get a nice fitting (see Supplementary Figure 16 above). It should be mentioned that the third Gaussian in the $FAPbI_3$ at ~6.5 cm^{-1} is only required for the fitting purpose and doesn't have any physical significance. On the contrary, the third Gaussian at ~28 cm^{-1} in the mixed halide PNCs may originate due to the FA molecule facing an anisotropic interaction with the inorganic cage as compared to the case of pure halide PNCs, as discussed in the main text.

It is to be mentioned that a very low intense noisy excited-state absorption (ESA) signals are present away from the bleach (e.g. >530 nm for $FAPbBr_3$) in our experiments which results low intense FFT finger print in these region (Supplementary Fig. 3 and 9). Previously Mathies *et. al.* observed similar but clear periodic oscillation in the excited state absorption feature in $MAPbI_3$ perovskites (and therefore, the corresponding FFT finger print) in a region away from the bleach (~200 meV redshift).[3] The physical origin of such ESA was ascribed to the formation of the excited state coherent phonons (mainly different Pb-X vibrational modes).[3] In the present work, we have studied three different samples grown and characterize at similar conditions and the conclusions are drawn based on the comparison of their different behavior under these similar conditions (and supported by theoretical calculations). Therefore, we believe such low intense ESA signals have minimal effect on our conclusions.

Supplementary Note 4: Details about the pump-probe data processing

The as collected raw data from the pump-probe experiments shows significant chirp along the probe wavelength (see Supplementary Fig. 2) as well as noisy weak modulations far from the bleach region (e.g. 530-560 nm for $FAPbBr_3$). Using Matlab, first a chirp-free data is generated, followed by the noisy modulation is removed (compare at Fig. 2c). The noisy modulation is removed considering the experimental data fluctuation which is strongly wavelength dependent (function of a polynomial of forth order). For example, at 560 nm the experimental data fluctuation is ~80% for $FAPbBr_3$ and any data have fluctuation >80% is removed. Similarly, at 530 nm the experimental data fluctuation is ~10% and the data having fluctuation >10 % is removed. The obtained chirp and noise-free data is used to generate the spectrum and time trace in the main manuscript (Fig. 2). The FFT is performed on the residual time trace, obtained after exponential subtraction of a specific time trace, mainly for the raw data for all the samples using Origin. The FFT spectrum remain unaltered upon performing on the processed chirp-free data.

Supplementary Note 5: The pump-probe stability

The pump and white light probe spectrum are shown in the Supplementary Fig. 17. To check the laser stability, the energy stability of the 400 nm pump and the attenuated continuum white light probe pulse at 600 nm are measured for 1 h duration and shown in Supplementary Fig. 18a, b. The corresponding standard deviation of the 400 nm pump intensity is 0.61 nJ (having average pump power 50.3 nJ, measured for 1 h i.e. ~1.2% energy fluctuation) and that of probe is 0.22 nJ (with average probe power 20.4 nJ, i.e. ~1.1% energy fluctuation). Furthermore, we have measured the white light probe pulse energy stability (attenuated) at various wavelengths (between 450 to 750 nm) for 60 sec each, in the span of total ~1 h duration, considered for extracting the FFT power spectra (Supplementary Fig. 18c). We have also reported the corresponding standard deviation of measured energy stability of the probe pulse for various wavelengths (Supplementary Fig. 18d). The standard deviation of measured energy stability of the probe pulse for various wavelengths varies between 160 to 180 pJ for the average energy between 20 to 21 nJ which is less than 1% probe energy fluctuation.

Supplementary References

1. Tong, Y.; Bladt, E.; Aygüler, M. F.; Manzi, A.; Milowska, K. Z.; Hintermayr, V. A.; Docampo, P.; Bals, S.; Urban, A. S.; Polavarapu, L. and Feldmann, J. Highly luminescent cesium lead halide perovskite nanocrystals with tunable composition and thickness by ultrasonication. *Angew. Chem. Int. Ed.* **55 (44),** 13887-13892 (2016**)**.
2. Huang, H.; Susha, A. S.; Kershaw, S. V.; Hung, T. F. and Rogach, A. L. Control of Emission Color of High Quantum Yield CH3NH3PbBr3 Perovskite Quantum Dots by Precipitation Temperature. *Adv. Sci.* **2 (9),** 1500194 (2015**)**.
3. Park, M., Neukirch, A. J., Reyes-Lillo, S. E., Lai, M., Ellis, S. R., Dietze, D., Neaton, J. B., Yang, P., Tretiak, S. and Mathies, R. A. Excited-state Vibrational Dynamics toward the Polaron in Methylammonium Lead Iodide Perovskite. *Nat. Commun.* **9,** 2525 (2018).
